# Frequency‐Magnitude Statistics of Laboratory Foreshocks Vary With Shear Velocity, Fault Slip Rate, and Shear Stress

**DOI:** 10.1029/2021JB022175

**Published:** 2021-11-12

**Authors:** David C. Bolton, Srisharan Shreedharan, Jacques Rivière, Chris Marone

**Affiliations:** ^1^ University of Texas Institute for Geophysics Austin TX USA; ^2^ Department of Engineering Science and Mechanics Pennsylvania State University University Park PA USA; ^3^ Dipartimento di Scienze della Terra La Sapienza Università di Roma Rome Italy

## Abstract

Understanding the temporal evolution of foreshocks and their relation to earthquake nucleation is important for earthquake early warning systems, earthquake hazard assessment, and earthquake physics. Laboratory experiments on intact rock and rough fractures have demonstrated that the number and size of acoustic emission (AE) events increase and that the Gutenberg‐Richter *b*‐value decreases prior to coseismic failure. However, for lab fault zones of finite width, where shear occurs within gouge, the physical processes that dictate temporal variations in frequency‐magnitude (*F*/*M*) statistics of lab foreshocks are unclear. Here, we report on a series of laboratory experiments to illuminate the physical processes that govern temporal variations in *b*‐value and AE size. We record AE data continuously for hundreds of lab seismic cycles and report *F*/*M* statistics. Our foreshock catalogs include cases where *F*/*M* data are not exponentially distributed, but we retain the concept of *b*‐value for comparison with other works. We find that *b*‐value decreases as the fault approaches failure, consistent with previous works. We also find that *b*‐value scales inversely with shear velocity and fault slip rate, suggesting that fault slip acceleration during earthquake nucleation could impact foreshock *F*/*M* statistics. We propose that fault zone dilation and grain mobilization have a strong influence on foreshock magnitude. Fault dilation at higher shearing rates increases porosity and results in larger foreshocks and smaller *b*‐values. Our observations suggest that lab earthquakes are preceded by a preparatory nucleation phase with systematic variations in AE and fault zone properties.

## Key Points

- Preseismic acoustic emissions represent foreshocks to lab earthquakes and evolve systematically during the lab seismic cycle
- *b*‐value of lab foreshocks decreases with shear velocity, fault slip rate, and shear stress
- Fault zone porosity and grain mobilization act in concert to produce larger foreshocks at higher shearing rates and fault slip rates

## 1 Introduction

Earthquake forecasting has been a fundamental goal of seismology for over a century (Bakun et al., 2005; Bernard et al., 1997; Crampin et al., 1984; Milne, 1899; Pritchard et al., 2020; Rikitake, 1968; Scholz et al., 1973; Whitcomb et al., 1973). The inability to accurately predict the location and timing of an impending earthquake is, in part, due to a poor understanding of earthquake nucleation. In particular, it is unclear how earthquake nucleation is linked to spatiotemporal changes in preseismic activity (e.g., foreshocks). Foreshocks are often considered a manifestation of earthquake nucleation and therefore identifying how foreshocks evolve in space and time could provide important insight for the physics of earthquake nucleation (Abercrombie et al., 1995; Chen & Shearer, 2013; Dodge et al., 1996; Ellsworth & Bulut, 2018; Kato et al., 2016; Mignan, 2014; Ohnaka, 1992, 1993; Yoon et al., 2019). Foreshock patterns in nature can be challenging to identify due to sparseness in seismicity and/or a lack of network coverage (Bakun et al., 2005). However, there are well documented examples of increased foreshock activity prior to the mainshock (Bouchon et al., 2013; Brodsky & Lay, 2014; Ellsworth & Bulut, 2018; Gulia & Wiemer, 2019; Gulia et al., 2016; Kato et al., 2016; Nanjo et al., 2012; Papadopoulos et al., 2010; Trugman & Ross, 2019; van den Ende & Ampuero, 2020; Wyss & Lee, 1973; Yoon et al., 2019). In some cases, the Gutenberg‐Richter *b*‐value decreases prior to the mainshock (Gulia et al., 2016; Nanjo et al., 2012); implying that foreshock magnitude increases systematically as the fault approaches failure.

There are also cases where mainshocks are not preceded by any form of seismic precursor, and these include examples with dense station coverage (Bakun et al., 2005). However, the absence of foreshocks could simply be due to issues with the development of earthquake catalogs. Ideally, earthquake catalogs should span several orders of magnitude and be complete down to low magnitudes (e.g., McBrearty et al., 2019; Ross et al., 2019; Walter et al., 2015). Earthquake catalogs often implement a thresholding scheme and/or some other constraint where events below a certain magnitude are discarded. However, this can lead to misguided conclusions about how foreshock patterns evolve in space and time. For instance, Trugman and Ross (2019) implemented a template matching approach (Quake‐Template‐Matching or QTM) and demonstrated that foreshock sequences may be more common than previously thought (see also van den Ende & Ampuero, 2020). This observation was driven by the fact that the QTM catalog was able to lower the magnitude of completeness well below that of standard catalogs. But the fact that foreshocks are not observed universally raises a fundamental question: what are the physical processes that control foreshock activity and why do some earthquakes appear to occur without a progressive failure process that includes foreshocks?

Laboratory experiments coupled with acoustic monitoring provide high‐resolution measurements of fault zone properties and acoustic activity throughout the lab seismic cycle. Therefore, they provide a unique opportunity to study foreshock dynamics in tandem with earthquake nucleation processes. Previous works have routinely documented precursory slip and seismic precursors prior to lab earthquakes (Acosta et al., 2019; Bolton et al., 2019, 2020; T. H. W. Goebel et al., 2013, 2015; Jiang et al., 2017; Kaproth & Marone, 2013; D. Lockner et al., 1991; D. A. Lockner et al., 1977; Lubbers et al., 2018; Main et al., 1989; McLaskey & Kilgore, 2013; McLaskey & Lockner, 2014; Ohnaka & Mogi, 1982; Passelègue et al., 2017; Renard et al., 2017, 2018; Rivière et al., 2018; Sammonds et al., 1992; Scholz, 1968a, 1968b; Scuderi et al., 2016; Shreedharan et al., 2020, 2021; Thompson et al., 2009; Tinti et al., 2016; Weeks et al., 1978). Many of these studies demonstrate that the event rate (number of events per unit time or slip) and magnitude increase as failure approaches. However, despite the robustness of this observation, it is not universally clear what physical processes allow acoustic emissions (AEs) to become bigger as failure approaches.

In the laboratory and in the field, foreshock sequences are often studied in terms of the Gutenberg‐Richter *b*‐value (Gutenberg & Richter, 1944):

(1) $${\text{log}}_{10}\left(N\right)=a-bM$$

where *N* is the number of events greater than or equal to magnitude *M*, “*a*” is a measure of seismic activity and “*b*,” referred to as the *b*‐value, describes the *F*/*M* distribution. It has long been known that *b*‐value decreases prior to failure of intact rock specimens (Scholz, 1968a) and prior to lab earthquakes (T. H. W. Goebel et al., 2013; Main et al., 1989; Weeks et al., 1978). Rock fracture experiments indicate that seismic events become bigger as time to failure decreases because stress increases and microfractures coalesce (Scholz, 1968a). Numerous studies have documented and validated the claim that *b*‐value and stress state are inversely related (T. H. W. Goebel et al., 2013; Gulia & Wiemer, 2019; Mori & Abercrombie, 1997; Nanjo, 2020; Rivière et al., 2018; Scholz, 2015; Schorlemmer et al., 2005; Spada et al., 2013; Wiemer & Wyss, 1997). However, it is not clear if shear stress alone is responsible for the temporal changes in *b*‐value that occur throughout the seismic cycle. Other possibilities include spatiotemporal variations in fault slip rate, fault zone dilation, stressing rate, and fault roughness (T. H. Goebel et al., 2017; McLaskey & Kilgore, 2013; Sammonds et al., 1992). Furthermore, several laboratory studies have demonstrated that AEs become more frequent and larger under boundary conditions that are well below the failure strength (Bolton et al., 2020; Hulbert et al., 2019; Jiang et al., 2017; Rivière et al., 2018; Rouet‐Leduc et al., 2018). Hence, it is possible that microfracturing plays a minor role in these experiments and that foreshock activity is dictated by other grain scale processes, such as the rupturing (i.e., sliding) of contact junctions. In this case, the size, strength, and number of contact junctions breaking per unit slip could play a fundamental role in regulating spatiotemporal properties of AE activity (e.g., Bolton et al., 2020; Mair et al., 2007; Yabe, 2002; Yabe et al., 2003).

It is also important to note that the decrease in *b*‐value observed in many laboratory studies occurs during inelastic loading where shear stress and fault slip rate are highly coupled (e.g., Dresen et al., 2020). Hence, without isolating these variables, it is not immediately clear which variable drives AE activity in lab experiments. Isolating the effects of shear stress and fault slip rate can be achieved experimentally by conducting shear stress oscillation experiments (e.g., Shreedharan et al., 2021) at stresses below the shear strength. Note that in these experiments the fault does not undergo periodic stick‐slip failure; instead, the shear stress is systematically modulated about a mean value that is just below the fault strength. Thus, the fault slip rate is zero and only the shear stress on the fault changes throughout the course of the oscillation. Hence, combining both types of experiments can isolate the effects of shear stress on *F*/*M* statistics of AEs, allowing for a more robust understanding of the causal processes that drive AE activity.

Here, we use laboratory friction experiments to document high‐resolution temporal characteristics of *F*/*M* statistics prior to stick‐slip failure. Experiments were conducted on simulated fault gouge over a wide range of conditions (Table 1). *F*/*M* statistics of AEs were derived from event catalogs and we performed an extensive set of sensitivity analysis on our event detection procedure. We record continuous AE data and we corroborate results from the earthquake catalogs by analyzing the continuous acoustic data. Our results are consistent with previous studies showing that *b*‐value decreases prior to failure. We show that the reduction in *b*‐value is most significant when the shear stress is ≥60% of the fault strength. In addition, we show that *b*‐value and AE magnitude scale inversely with the fault slip velocity and shearing velocity.

**Table 1:** List of Experiments and Boundary Conditions

Experiments	Normal stress (MPa)	Shear velocity (µm/s)	Mean grain size (µm)	Layer thickness (mm)
p5363	5	0.3–100	126.5	3.0
p5349	5	21	126.5	3.0
p5357	5	21	126.5	6.0
p5364	5	21	450	3.0
p5365	5	21	1100	3.0
p4348	9	2–60	10.5	3.0

## 2 Friction Experiments and Acoustic Emission Monitoring

**Figure 1:**
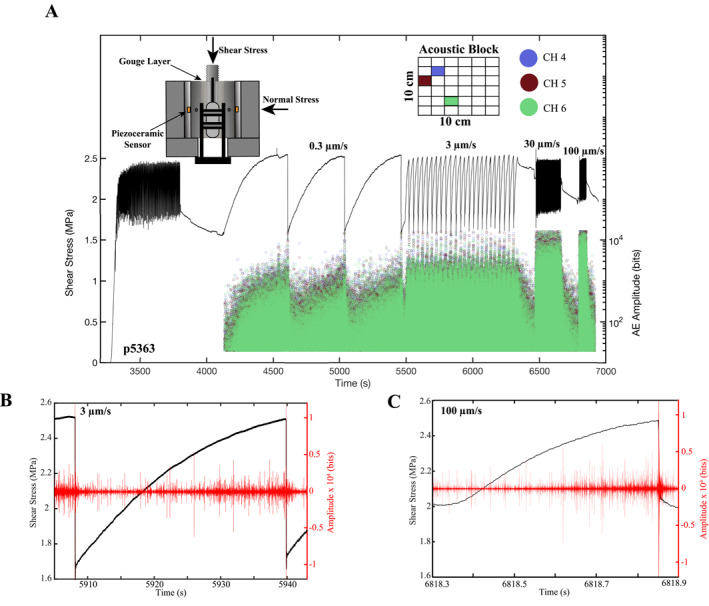
(a) Shear stress and acoustic emission (AE) amplitude plotted as a function of time. Open symbols represent AE amplitudes and are color coded according to the sensor they were detected on. Top left inset shows double‐direct shear configuration with acoustic blocks. Top right inset shows 2D schematic of acoustic block and the locations of the three sensors used in this study. Acoustic amplitude increases throughout the seismic cycle and larger AEs nucleate during the interseismic period for higher shear velocities. (b, c) Shear stress and continuous AE data plotted as a function of time for one entire seismic cycle at 3 and 100 μm/s. Spikes in the continuous acoustic data are AEs that are cataloged according to their peak amplitude and plotted in panel (a).

We report on laboratory shear experiments conducted on soda‐lime glass beads and quartz powder (Min‐U‐Sil) in a servo‐hydraulic testing machine using the double‐direct shear (DDS) configuration (Figure 1 inset). Glass beads are commonly used as synthetic fault gouge because their frictional and seismic properties are highly reproducible and include both the time‐ and slip rate‐dependent friction effects observed for geologic materials (Anthony & Marone, 2005; Jiang et al., 2017; Mair et al., 2002; Marone et al., 2008; Rivière et al., 2018; Scuderi et al., 2014, 2015). We shear two fault zones between three roughened steel forcing blocks. The surfaces of the forcing blocks are rough (triangular grooves that are 0.8 mm deep and 1 mm in width) to eliminate slip at the fault zone boundary. We studied shear velocities from 0.3 to 100 μm/s, and a range of grain sizes and fault zone thicknesses (Table 1). All experiments conducted on glass beads were run at constant fault normal stress of 5 MPa. Fault stresses and displacements were measured continuously at 1 kHz using strain‐gauge load cells and direct‐current displacement transformers (DCDTs). The loading velocity was prescribed at the central block of the DDS configuration (Figure 1). We also measured the true fault slip velocity with a DCDT mounted on the central shearing block and referenced to the base of the vertical load frame (Figure 1). Throughout the text, we refer to the load‐point velocity as the shearing velocity and the independently measured fault slip rate as such. To ensure reproducibility, all experiments were conducted at room temperature and 100% relative humidity (RH). Prior to each experiment, gouge layers were placed inside a plastic bag for 12–15 hr with a 1:2 ratio of sodium carbonate to water solution. During the runs, samples were isolated with a plastic membrane to maintain 100% RH conditions. Changes in RH conditions are known to greatly affect frictional properties of granular media (Frye & Marone, 2002; Scuderi et al., 2014), hence keeping it constant helps ensure reproducibility across experiments.

AE data were recorded throughout the experiment using a 15‐bit Verasonics data acquisition system. AE data were recorded continuously at 4 MHz using broadband (∼0.0001–2 MHz) piezoceramic sensors (6.35 mm diameter and 4 mm thick). The sensors are located 22 mm from the edge of the fault zone at the base of blind holes in steel loading platens (Figure 1 inset; Bolton et al., 2019; Rivière et al., 2018). We recorded data from a total of six sensors located on both sides of the DDS configuration. Here, we report data from three sensors located on the left side of the DDS assembly (Figure 1 inset).

### 2.1 Acoustic Emission Catalog Development and *b*‐value Calculation

We derive frequency–magnitude statistics of AEs using a thresholding procedure to scan through the continuous AE signal and catalog events according to their peak amplitude. Our method derives from that of Rivière et al. (2018) with extensive modifications and sensitivity analysis to evaluate the effect of the thresholding parameters on event detection (see Supporting Information S1 for additional details). Our detection algorithm uses four thresholding parameters. First, we compute the envelope of the continuous AE signal and smooth the envelope using a moving average, *A*_Env_. We then scan through the continuous data and detect a set of candidate AEs based on a minimum interevent time threshold, *T*_min_, and minimum amplitude threshold, *A*_min_. *T*_min_ ensures that two adjacent AEs are separated in time by a minimum value and *A*_min_ is set right above the noise level. In theory, there is no reason why two adjacent AEs must be separated in time by *T*_min_; however, imposing this constraint helps ensure that the same event is not picked repeatedly. To determine *T*_min_, we manually compute the duration of several hundred AEs and use the median of this distribution as *T*_min_ (Figure S1 in Supporting Information S1). In addition, we impose a ring‐down‐time (RDT) threshold, *T*_RDT_, to avoid picking the same event repeatedly immediately after the peak amplitude and to account for sensor resonance. The objective of *T*_RDT_ is to ensure that AEs are not detected within the coda of a former event. *T*_RDT_ is imposed after the algorithm has identified a set of candidate events based on *A*_env_, *A*_min_, and *T*_min_. Once the algorithm has identified a set of candidate events, we apply *T*_RDT_ through the following procedure. For a candidate event *A*_*j*_, we apply *T*_RDT_ to the previous five events. If the amplitude of *A*_*j*_ lies above the ring‐down time curves of the previous five events, then we catalog the peak amplitude and time of event *A*_*j*_ (Figure S2 in Supporting Information S1). More specifically, we catalog event *A*_*j*_ if it meets the following criteria:

(2) $$A_{j\;}>A_{j-i}\times \text{exp}\left[-\frac{\left(t_{j}-\;t_{j-i}\right)}{T_{\text{RDT}}}\right]\;\;\text{where}\;,i=\left[1,\;2,\;\text{…},\;5\right]$$

where $t_{j}$ and $t_{j-i}$ are the time stamps associated with the candidate event and the previous five events, respectively. We determine *T*_RDT_ by computing the ring‐down times of ∼100 randomly picked AEs. We then compute the median value of this distribution and set this equal to *T*_RDT_ (Figure S1 in Supporting Information S1). Therefore, we use one RDT to model all the AEs detected. We recognize that this approach may not optimally model all the events (Figure S2 in Supporting Information S1) because the RDT of an event can change depending upon the source mechanism associated with that particular event. A more robust approach would involve using a multivalued RDT to model different “families of AEs.” However, this is beyond the scope of the current study and we use other techniques to verify our results, as described below.

Once the event criteria are set by the four thresholding parameters, we scan the continuous data for each channel and catalog the peak AE amplitudes and times (see Figure S3 in Supporting Information S1). Note, the event detection procedure treats each channel independently. For Experiment p5363, we used the following thresholding parameters: *A*_Env_: five data points, *A*_min_: 20 (bits), *T*_min_: 131 µs, and RDT: 93 µs. We show results from an extensive sensitivity analysis for each thresholding parameter and its impact on *b*‐value in Supporting Information S1. Our analysis indicates that the thresholding parameters do not have a significant impact on the temporal changes in *b*‐value (Figure S4 in Supporting Information S1).

We use a moving window on the cataloged events to compute the Gutenberg–Richter *b*‐value. *b*‐values were estimated using a maximum‐likelihood approach (Aki, 1965):

(3) $$b=\;\frac{{\text{log}}_{10}\left(e\right)}{\left(\bar{M}-M_{c}\right)}$$

where *M*_c_ is the magnitude of completeness and $\bar{M}$ is the average magnitude above *M*_*c*_, and *e* = exp(1). Similar to previous field and laboratory studies, we compute *b*‐values using a constant number of events to ensure that each *b*‐value is statistically similar (T. H. W. Goebel et al., 2013, 2015; Gulia et al., 2020; Herrmann et al., 2019; Nanjo, 2020; Nanjo et al., 2012; Ojala et al., 2004; Rivière et al., 2018; Tormann et al., 2013). To determine the number of events for each *b*‐value calculation (*N*_AE_), we first compute the cumulative number of events across multiple seismic cycles and for every channel (Figure S5 in Supporting Information S1). We focus here on the interseismic period and thus consider events from the interval defined by the minimum shear stress and the peak stress of a given seismic cycle. To compute *N*_AE_, we average the cumulative number of events across multiple slip cycles for each channel and take *N*_AE_ as 10% of this value. Because the recurrence interval scales inversely with shear velocity, the cumulative number of events per seismic cycle, and thus *N*_AE_ is larger at lower shearing rates (Figure S5 in Supporting Information S1). Increasing or decreasing *N*_AE_ has a trivial effect on the results; decreasing this number simply increases the number of *b*‐value calculations per seismic cycle, and therefore the temporal resolution.

It is important to acknowledge that our *F*/*M* distributions do not strictly follow an exponential relation. However, to compare our work to previous studies, we stick with convention and refer to the slope of the *F*/*M* curves as the laboratory *b*‐value. Accurate estimations of *M*_c_ are essential for reliable *b*‐value calculations (Herrmann & Marzocchi, 2021). In this study, we determine *M*_c_ from the peak of the noncumulative distribution (e.g., Woessner & Wiemer, 2005). We acknowledge that this method may not be suitable for all *F*/*M* curves, particularly those that contain some degree of curvature (e.g., 100 μm/s; Figure S6 in Supporting Information S1). In such cases, the peaks of the noncumulative distribution may not accurately represent the best *M*_c_ value. However, other standard approaches for estimating *M*_c_ (e.g., goodness‐to‐fit) for data at 100 μm/s place *M*_c_ at a slightly higher value (*M* > 2.0) which results in estimating *b*‐values based on the tails of *F*/*M* distributions, where few events exist (Wiemer & Wyss, 2000). All in all, the main complexity here is not in the methods used to compute *M*_c_, but rather in the fact that not all of the *F*/*M* curves exhibit a power law scaling.

We plot the noncumulative and cumulative distribution of AEs for a single moving window at different locations in the seismic cycle for data at 0.3 and 100 μm/s in Figure S6 in Supporting Information S1. At 0.3 μm/s, *M*_c_ is ∼1.35 and does not change as a function of position within the seismic cycle. In contrast, at 100 μm/s, *M*_c_ is higher and increases as the fault approaches failure. Because we use a moving window approach to compute *b*‐values, we could let *M*_c_ vary for each moving window and for each shearing velocity. However, we argue that this approach would result in an inconsistent comparison of *b*‐values as a function of shearing velocity and position within the seismic cycle because of the different magnitude ranges (Figure S6 in Supporting Information S1). To circumvent this issue and to ensure a more reliable comparison of *b*‐value as a function of shearing velocity, we select a “global *M*_c_” to compute *b*‐values. That is, we use a single *M*_c_ value to compute *b*‐values across the entire seismic cycle and for each shearing velocity. Our data show that the highest shear velocity (100 μm/s) produces the highest *M*_c_. Therefore, we select our “global” *M*_c_ such that it is ≥*M*_c_ at 100 μm/s. To determine our “global *M*_c_,” we use focus on data at 100 μm/s and compute *M*_c_ (peak of the noncumulative distribution) at multiple locations within the stick‐slip cycle for each channel. For a given channel, we then average the *M*_c_ values across the multiple locations and set this average value equal to the “global *M*_c_.” This procedure results in a *M*_c_ of 2.15, 2.16, and 2.02 for channels 4–6, respectively. We then estimate *b*‐values for each channel using its corresponding *M*_c_ value. Here, *M* is defined as logarithm of the peak amplitude.

## 3 Results

Experiment p5363 began with a run‐in shear displacement of 5 mm after which the shear velocity was decreased to 0.3 μm/s and then subsequently increased in steps to 100 μm/s (Figure 1). The size of the slip events and the recurrence interval of the seismic cycles decrease with increasing shear velocity (Figure 1). AE amplitude also increases with shearing velocity, consistent with previous works (Jiang et al., 2017; Ojala et al., 2004; Yabe et al., 2003). Our system records acoustic data continuously, so the AE amplitudes plotted in Figure 1a are derived from the continuous AE data (e.g., Figures 1b and 1c). The spikes in the continuous AE records correspond to discrete AEs that are detected using our cataloging procedure described above.

We analyze AE event rates across the interseismic period using a moving window. The width of each window corresponds to 10% of the recurrence interval of the seismic cycle and each window overlaps the previous window by 99%. In addition, windows are normalized by the load‐point displacement, to account for the expectation that more AEs could occur if the fault slips more (e.g., Mair et al., 2007).

**Figure 2:**
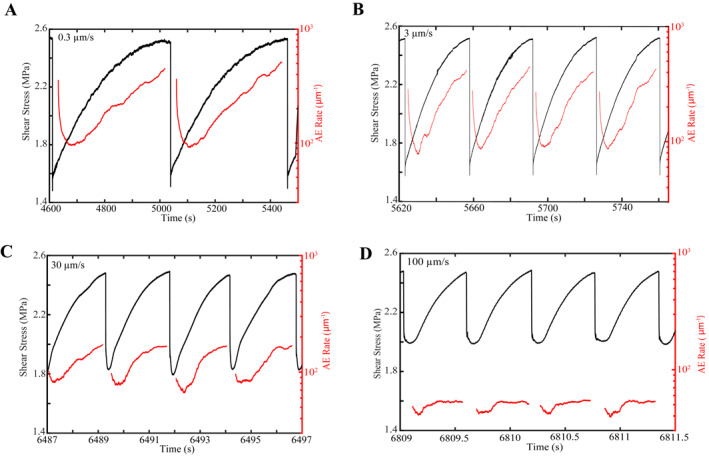
(a–d) Shear stress and AE rate (per unit displacement) as a function of time for different shear velocities explored in this study (see Figure 1). AE rate is calculated using a time window whose width corresponds to 10% of the recurrence interval of the seismic cycle. For each window, we count how many events were detected and normalize each time window by the amount of slip displacement covered. Event rates are high after a failure event, decrease to a minimum, and subsequently increase until coseismic failure.

AE event rates vary systematically during the lab seismic cycle. After a lab mainshock, the AE event rate decreases, reaches a minimum, and then increases continuously until the next mainshock (Figure 2). The postseismic reduction in event rate appears to scale with the size of the previous mainshock. The absolute value in AE event rate decreases modestly with increasing shear velocity for low shearing rates (0.3–3.0 μm/s) and is more pronounced for shearing rates ≥30 μm/s (Figure 2). The evolution of event rate over the seismic cycle also varies with loading velocity. The AE event rate increases significantly prior to failure for slower shearing rates. In contrast, at higher shearing rates, the event rate appears to saturate prior to failure (Figure 2).

**Figure 3:**
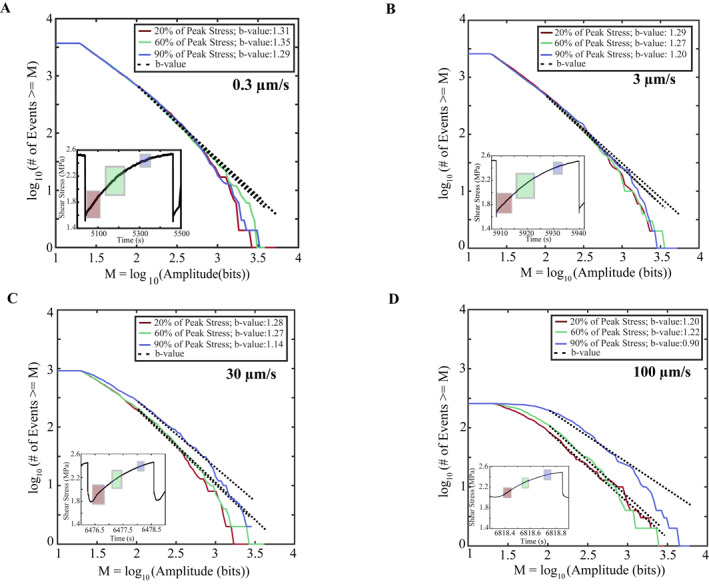
(a–d) Frequency‐magnitude plots are shown for each shear velocity at different locations in the seismic cycle. *F*/*M* plots represent AE statistics derived from our cataloging approach from Experiment p5363. Curves are color coded according to their location within the seismic cycle and the black dashed line represents the magnitude range used to compute *b*‐values. Note, each inset shows the specific seismic cycle from which the *F*/*M* curves are derived from. The color coded squares in the inset correspond to the time window associated with each *F*/*M* curve. *F*/*M* curves are plotted using a constant number of events, and thus, windows vary in time at each location within the seismic cycle and become smaller as time to failure approaches zero due to higher event rates (see Figure 2). *b*‐value decreases as failure approaches and scales inversely with shear velocity.

**Figure 4:**
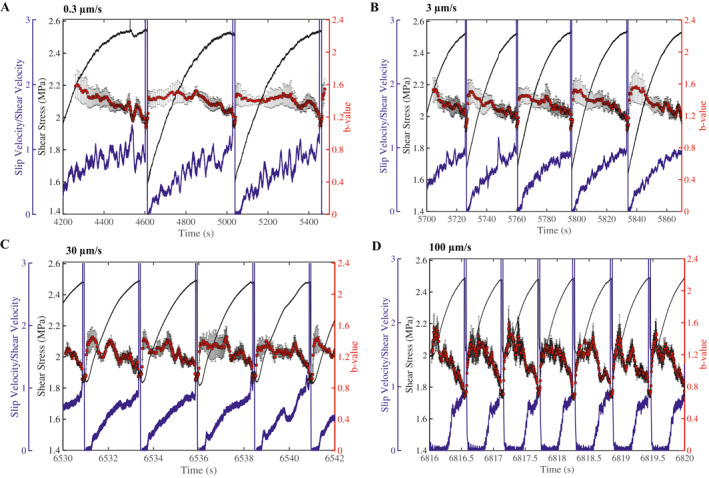
(a–d) Shear stress, fault slip velocity, and *b*‐value as a function of time for different shear velocities. *b*‐values are averaged across three channels and the error bars represent one standard deviation among the channels. The preseismic changes in fault slip rate show that the fault unlocks very early on in the seismic cycle and increases continuously until coseismic failure. *b*‐value decreases systematically throughout the seismic cycle for each shearing velocity.

**Figure 5:**
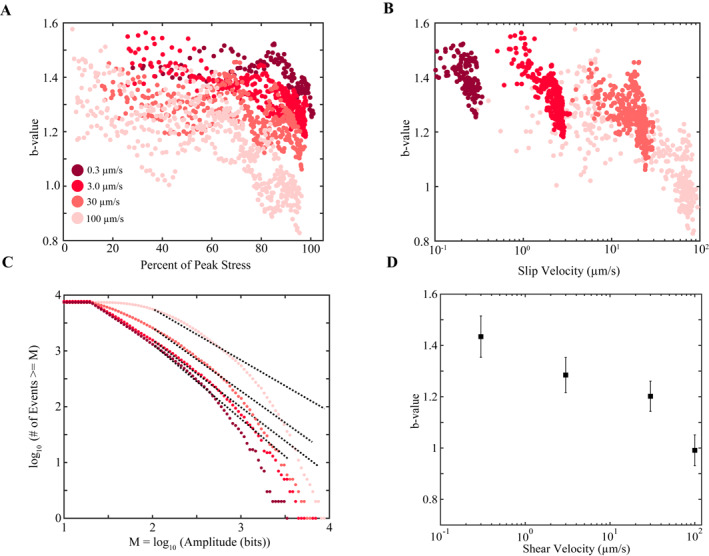
(a) *b*‐value plotted as a function of shear stress. Note, *b*‐values correspond to the same data plotted in Figure 4. *b*‐value scales inversely with shear stress once the fault surpasses ∼60% of its peak stress and is inversely correlated with shear velocity. (b) *b*‐value versus true fault slip velocity. Note, the strong correlation between *b*‐value and slip rate for a given shearing velocity. (c) *F*/*M* statistics derived from stacking multiple *F*/*M* curves at 90% of the peak stress, resulting in a total of ∼7,400 AEs for each *F*/*M* curve. (d) *b*‐value scales inversely with shear velocity for data at 90% of the peak stress. Note, *b*‐values are estimated from *F*/*M* curves in (c).

**Figure 6:**
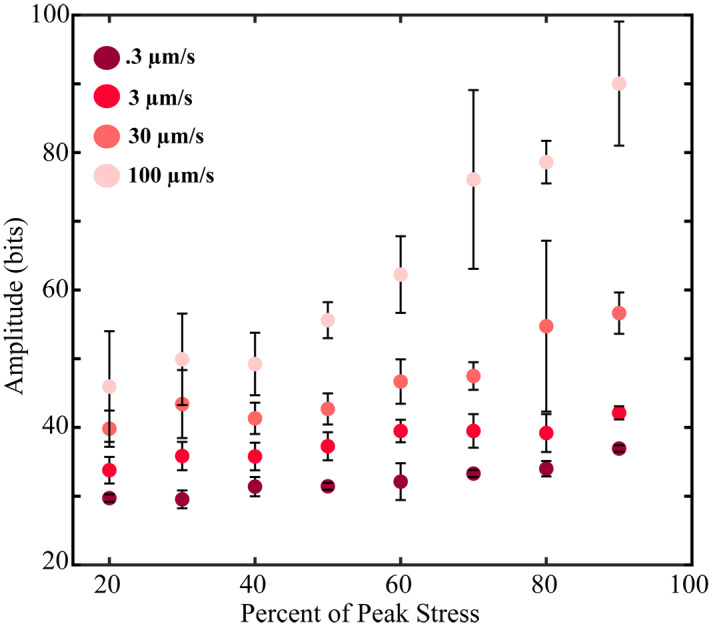
Mean acoustic amplitude derived from the continuous AE data. Amplitudes are averaged across all the slip cycles shown in Figure 4 for a given shear velocity and location within the seismic cycle. 1 μm windows are used to compute mean values. AE amplitude increases as the fault approaches failure and scales inversely with the shear velocity.

**Figure 7:**
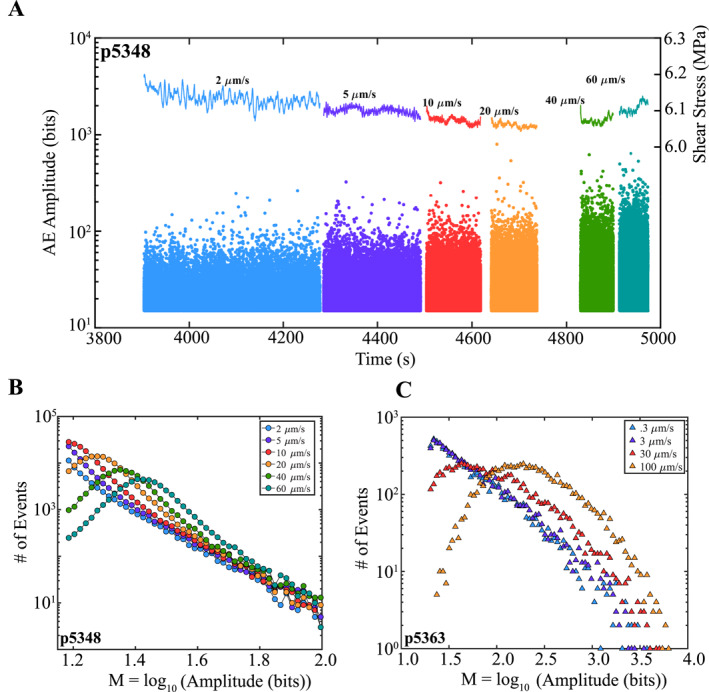
(a) AE amplitude and shear stress plotted as a function of time for a stable sliding friction experiment (p5348). AE amplitude increases with shearing velocity. (b) Histogram of AE amplitudes for 500 μm windows. Higher shearing velocities show a net increase in larger events (*M* ≥ 1.42) relative to lower shearing rates. (c) Histogram of AE amplitudes derived from stacking multiple seismic cycles at 85% of the peak stress, resulting in ∼7,400 AEs for each shear velocity (stick‐slip experiment p5363). *F*/*M* data show an increase in bigger events relative to smaller events at higher shearing rates.

We document temporal changes in *b*‐value as a function of position in the seismic cycle and as a function of shearing velocity (Figures 3, 4, 5, 6, 7). *F*/*M* curves vary systematically with shear velocity and position within the seismic cycle (Figure 3). The data demonstrate that the *b*‐value (black dotted line) decreases as the fault approaches failure. This can be seen by noting how the *F*/*M* curves become vertically offset at larger magnitudes as failure approaches (Figure 3). The changes in *b*‐value are subtle during the early stages of the seismic cycle. The *b*‐value only begins to decrease significantly once the fault has surpassed ≥60% of its peak stress. In addition to the stress dependence of *b*‐value, we also find that *b*‐value depends on the shearing velocity. This can be seen clearly by noting how the *F*/*M* curves become more offset as the fault transitions from 60% to 90% of the peak stress for different shearing velocities (Figure 3). At 0.3 μm/s, the *F*/*M* curves are nearly identically at 60% and 90% of the peak stress (implying that the *b*‐values are similar). In contrast, at 100 μm/s, the offset between the *F*/*M* curves at 60% and 90% peak stress is significant (Figure 3).

Our data also show that *b*‐value varies with shear stress and fault slip velocity (Figure 4). To characterize uncertainty in our *b*‐value measurements, we plot the average *b*‐value for three channels (see Figure 1); error bars represent one standard deviation among the channels. To compute the temporal changes in *b*‐value, we use a moving window on the cataloged AEs (Figures 3 and 5). The size of each window contains a constant number of events (see methods) and each window overlaps the previous window by 90%. Fault slip velocity is derived from the on‐board DCDT (Figure 1a). Generally, the fault unlocks early in the seismic cycle and the fault slip rate increases continuously until coseismic failure. *b*‐value decreases slightly during the early stages of the seismic cycle and more significantly once the fault is closer to failure (Figure 4), consistent with previous works (T. H. W. Goebel et al., 2013, 2015; Rivière et al., 2018; Sammonds et al., 1992; Scholz, 1968a, 1968b; Weeks et al., 1978).

To assess the shear stress, shear velocity, and fault slip rate dependence of *b*‐value more clearly, we plot data from Figure 4 as a function of location within the seismic cycle and fault slip velocity (Figures 5a and 5b). This shows that *b*‐value (a) decreases significantly once the fault reaches ∼60% of its peak stress and (b) scales inversely with the shearing velocity and fault slip rate. To investigate the far‐field shear velocity dependence of *b*‐value further, we stack multiple *F*/*M* curves located at 90% of the peak stress and estimate their *b*‐values for different shear velocities (Figures 5c and 5d). The data clearly indicate that higher shearing rates produce a net increase in larger AEs relative to smaller AEs.

The data of Figures 3, 4, 5 demonstrate that *b*‐value is lower near failure and scales inversely with fault slip velocity and shearing velocity. However, these results are based on a catalog of AEs and it is possible that the detection algorithm (see Section 2.1) misses some of the smaller AEs. In fact, our cataloged data show that *M*_c_ increases as time to failure decreases for data at 30 and 100 μm/s (Figure 3 and Figure S6 in Supporting Information S1). In theory, if all events were detected, we would not expect *M*_c_ to shift as a function of position in the seismic cycle. A potential culprit could be the thresholding parameters used in catalog construction. In particular, it is possible that we miss lower magnitude events as failure approaches because the events occur quasi‐simultaneously and the ring‐down of larger amplitude events masks smaller events. Therefore, the reduction in *b*‐value prior to failure could simply be a catalog completeness issue.

To circumvent common issues associated with cataloging, we complement our catalog‐based results by computing mean values of the continuous acoustic signal (Figure 6). Figure 6 shows the mean acoustic signal amplitude as a function of normalized shear stress for multiple seismic cycles. Here, we use a 1‐μm window to compute mean amplitudes across multiple locations in the seismic cycle from the stick‐slip cycles shown in Figure 4. These data show that the mean AE amplitude increases as failure approaches and that it scales inversely with the shearing velocity. Note, the mean values lack information regarding event distributions, and thus, we are unable to directly compare these data to our *b*‐value estimates. Nevertheless, the mean values help validate our *b*‐value observations and demonstrate that our cataloging procedure does not lead to misguided conclusions about how *b*‐value evolves throughout the seismic cycle.

In addition to verifying the trends observed in Figures 3, 4, 5, we also verified the velocity dependence of AE size by analyzing *F*/*M* statistics of AEs generated during a stable sliding experiment (p5348; Figure 7a). We then compared these data to *F*/*M* statistics derived from Experiment p5363 (Figures 1, 2, 3, 4, 5, 6). In Experiment p5348, we sheared quartz powder (Min‐U‐Sil) under a constant normal load of 9 MPa and swept through a range of shearing velocities from 2 to 60 μm/s. The boundary conditions of Experiment p5348 permitted stable frictional sliding (i.e., no stick‐slips). The velocity dependence of AE size is clear (Figure 7a); higher shearing rates produce bigger AEs. We further verify these results by using a 500‐μm window to compute noncumulative *F*/*M* distributions of AEs at each shearing velocity (Figure 7b). *F*/*M* distributions indicate that higher shearing rates result in a net increase in larger magnitude events (Figure 7b).

Experiment p5363 (stick‐slip experiment; Figure 1) shows similar event distributions compared to the stable sliding experiment (Figure 7c). Here, we plot results after stacking multiple seismic cycles at 85% of the peak stress for each shear velocity. Again, higher shearing rates show a systematic increase in larger magnitude events and are deficient in smaller magnitude events (Figure 7c). Note, that the shape of the *F*/*M* curves is similar for both the stable sliding experiment and stick‐slip experiment. That is, at low shear rates, the noncumulative event distributions scale ∼linearly with magnitude, whereas at higher shearing rates, the *F*/*M* curves approach a Gaussian‐like shape.

To conclude, all of our *F*/*M* curves may not follow a strict exponential relationship and this could lead one to question the validity of our *b*‐value results (e.g., Herrmann & Marzocchi, 2021; van der Elst, 2021). However, the purpose of this work is to demonstrate that preseismic AEs (i.e., foreshocks) are modulated by shear velocity, shear stress, and fault slip rate. The velocity dependence of AE size can be seen in the raw data in Figure 1 and in the noncumulative distributions in Figure 7. The rate dependence of AE size holds true regardless of whether or not our *F*/*M* curves follow a strict exponential relationship or if it is theoretically correct to refer to the slopes of the *F*/*M* curves as the “*b*‐value.” We use *b*‐value as a metric to quantify AE size and to connect our observations to previous studies.

## 4 Discussion

Connecting temporal changes in foreshock sequences to the physical properties of fault zones is a fundamental problem in earthquake seismology (e.g., Frankel, 1991; King, 1983; Scholz, 1968a; Weeks et al., 1978; Wiemer & Wyss, 1997). The connection between seismic activity and fault zone processes is key to understanding the physics of earthquake nucleation and improving earthquake early warning systems and forecasting (Abercrombie & Mori, 1996; Abercrombie et al., 1995; Chen & Shearer, 2013; Dodge et al., 1996; Ellsworth & Beroza, 1995; Kato et al., 2016; Lockner et al., 1991; McLaskey, 2019; Ohnaka, 1992, 1993, 2000). A plethora of laboratory studies, and several field studies, have demonstrated that the frequency and magnitude of foreshocks increase prior to failure (Bouchon et al., 2013; Brodsky & Lay, 2014; Chen & Shearer, 2013; Ellsworth & Bulut, 2018; T. H. W. Goebel et al., 2013; Gulia & Wiemer, 2019; Kato et al., 2016; McLaskey & Lockner, 2014; Nanjo et al., 2012; Papadopoulos et al., 2010; Rivière et al., 2018; Ruiz et al., 2014; Sammonds et al., 1992; Scholz, 1968a, 1968b; Trugman & Ross, 2019). However, the physical processes that cause earthquakes to become more frequent and larger as a mainshock approaches is unclear. Scholz (1968a) demonstrated that the magnitude of AEs for failure of intact rock is inversely related to the differential stress and he attributed this relationship to the formation and coalescence of microfractures. However, it is unknown how well this interpretation extrapolates to tectonic fault zones where failure may occur within breccia and fault gouge. Our data show that shear stress plays an important role in modulating *b*‐value, but we also see that *b*‐value scales inversely with fault slip velocity and shearing rate. Hence, there must be processes other than stress state that influence foreshock size.

### 4.1 Acoustic Emission Event Rates

Laboratory studies show that AE event rates increase systematically as failure approaches and are consistent with the temporal evolution of foreshock sequences in tectonic fault zones (Acosta et al., 2019; Amitrano, 2003; T. H. W. Goebel et al., 2015; McLaskey & Lockner, 2014; Mogi, 1962; Ojala et al., 2004; Sammonds et al., 1992; Scholz, 1968a, 1968b; Weeks et al., 1978). However, few studies have documented the velocity dependence of this process. Our data show that the event rate of lab foreshocks per unit fault slip scales inversely with shear velocity (Figure 2). However, the changes are largest at our highest loading rates (30 and 100 μm/s) and the increase in *M*_c_ with velocity could partially bias the AE rate evolution if more small events are missed at larger shearing velocities (Figure S7 in Supporting Information S1).

**Figure 8:**
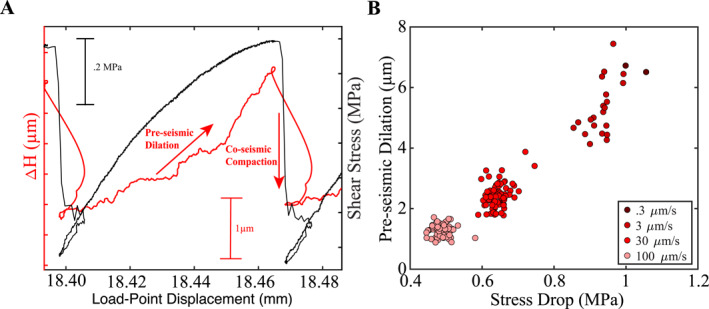
(a) Layer‐thickness and shear stress plotted for one seismic cycle. Preseismic dilation is computed as the change in layer‐thickness across the interseismic period. (b) Dilation plotted as a function of stress drop for each shearing velocity explored in Experiment p5363. Dilation scales systematically with stress drop and inversely with shearing velocity.

On the other hand, low event rates at higher velocities could arise from a true lack of smaller events at these shearing rates. For example, it is possible that preseismic fault zone dilation plays an important role in controlling the event rates (Figure 8). Our data indicate that preseismic fault dilation scales inversely with the far‐field shearing velocity, thus higher dilation at lower shearing rates could lead to more interparticle slip and rolling among grains, and as a result an increase in acoustic activity.

The temporal evolution in event rates across the seismic cycle is also worth mentioning. In particular, the postseismic reduction in event rate seems to scale with the stress drop of the previous slip event (Figure 2). This postseismic reduction in event rate could be a proxy for aftershock activity. It should be made clear that the reduction in event rate is not due to an artifact of the windowing procedure. The moving windows start at the beginning of the seismic cycle and do not include temporal information from the previous slip cycle. Furthermore, this reduction in AE activity is also captured in other higher‐order statistics of the AE signal, such as the acoustic energy (i.e., variance). The acoustic energy decreases following a slip event and shows a similar temporal evolution to the AE event rates (Bolton et al., 2020; Hulbert et al., 2019). Although it is not mentioned in these studies, the reduction in AE energy following the slip event could also be evidence of aftershock activity. In addition, these observations are consistent with 3D discrete element models that show elevated levels of kinetic energy and microslips following stick‐slip events (Ferdowsi et al., 2013).

### 4.2 Shear Stress, Fault Slip Rate, and Shearing Velocity Dependence of *F*/*M* Statistics

For a given shear velocity, the data from Figure 5a are strongly correlated with the true fault slip velocity (Figure 5b). To assess the shear velocity dependence on *b*‐value, we normalize the true fault slip velocity by the shearing velocity and replot the data from Figure 5b (Figure S8 in Supporting Information S1). The data show that *b*‐value scales inversely with normalized fault slip rate. Interestingly, the *b*‐values are still offset with respect to shearing velocity. Because the data do not collapse onto a single curve, this suggests that *b*‐value is modulated by both the shearing velocity and the fault slip rate. The shear velocity only affects the absolute values of *b*‐value and does not seem to have a significant effect on the temporal changes; if indeed the shearing velocity did have an effect on the temporal reduction in *b*, then we should expect to see a higher‐order effect superimposed on the data in Figure S8 in Supporting Information S1. Hence, these observations suggest that the inverse relationship between *b*‐value and shear velocity in Figure 5a is a shear rate effect, while the reduction in *b*‐value prior to coseismic failure is ultimately tied to the simultaneous and continuous increase in fault slip rate and shear stress.

In most laboratory stick‐slip experiments, shear stress and fault slip rate are highly coupled and increase continuously throughout the laboratory seismic cycle (Figure 4). In our experiments, this coupling could be due to the intrinsic, mechanical properties of glass beads and/or the fact that our experiments are conducted at low normal stresses (5 MPa). Thus, we decoupled the effects of fault slip rate from shear stress by conducting shear stress oscillation experiments under boundary conditions that resulted in zero fault slip (Figure S9 in Supporting Information S1). Prior to shear stress oscillations, faults were sheared for 10 mm at 21 μm/s, producing lab earthquakes as in Figure 1. Then, the shear stress was reduced to ∼50% of the peak stress. This limits the amount of fault creep and helps ensure that the fault slip rate was ∼0 during the shear stress oscillations. However, because the shear stress was reduced to 50% of the peak stress, these experiments are only compatible with the early stages (e.g., ≤50% of the peak stress) of our stick‐slip experiments. Nevertheless, for stresses below 50% of the peak stress, our data demonstrate that changes in shear stress on the fault alone do not induce changes in the *F*/*M* statistics of AEs (Figure S9 in Supporting Information S1). These results are consistent with those in Figure 5a, which show that *b*‐value changes are subtle in the early stages of the seismic cycle (≤60% of the peak stress). The data of Figure S9 in Supporting Information S1 shows that the shear stress does not affect *F*/*M* statistics early in the seismic cycle. At stresses above 50% of the peak stress, both shear stress and changes in fault slip rate impact *F*/*M* statistics of lab foreshocks.

### 4.3 A Micromechanical Model for the Velocity Dependence of AE Size and *b*‐value

At first glance, the inverse relationship between fault slip rate and shear velocity with *b*‐value may seem counterintuitive with respect to frictional healing processes. Basic concepts of time‐dependent frictional healing would predict stronger contacts and elevated friction at lowering shearing rates (Dieterich, 1972, 1978). Our data indeed show that stress drop decreases with increasing shear velocity, consistent with expectations for frictional aging and previous lab results (e.g., Karner & Marone, 2000). Moreover, our previous work establishes (a) that stress drop varies systematically with peak fault slip velocity of laboratory earthquakes, with slow events having smaller stress drop and (b) that coseismic acoustic energy release scales directly with stress drop (Bolton et al., 2020). Thus, the expectation that laboratory earthquakes with larger stress drop have larger coseismic acoustic amplitude is consistent with our data. However, we find that larger laboratory foreshocks nucleate at higher preseismic slip rates, so the underlying mechanism that modulates foreshock size seems to derive from something other than contact junction age and frictional healing.

At a simplistic level, the generation of AEs in granular fault gouge must arise from a combination of grain fracturing, grain sliding/rolling, and the breaking of force chains. Because our experiments were conducted at low normal stress (5 MPa), grain crushing and comminution are insignificant (Mair et al., 2002; Scuderi et al., 2015). Thus, AE generation in our experiments is likely driven by nondestructive grain scale processes, such as grain sliding and rolling, and shear of partially welded contact junctions. Previous laboratory studies have demonstrated that AEs/foreshocks have corner frequencies of a few hundred kilohertz and source dimensions of a few millimeter (McLaskey & Lockner, 2014; McLaskey & Yamashita, 2017). Therefore, it is reasonable to assume that a single AE represents the movement of multiple contact junctions, given that the size of a single contact junction ranges between 10–149 μm.

#### 4.3.1 Enhanced Porosity and Grain Mobilization as a Mechanism for the Velocity Dependence of AE Size and *b*‐value in Granular Fault Zones

**Figure 9:**
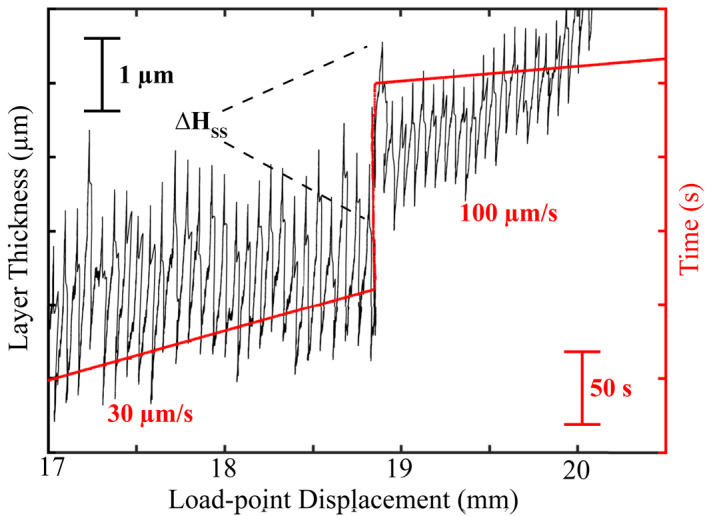
(a) Detrended layer‐thickness and time plotted as a function of load‐point displacement (p5363). Upon a step increase in load‐point velocity, the steady‐state layer‐thickness (Δ*H*_SS_) increases instantaneously and semipermanently.

**Figure 10:**
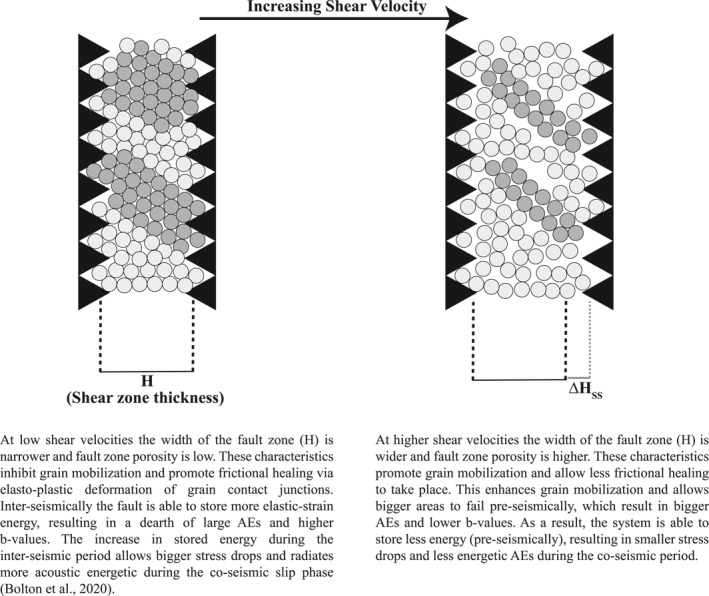
A 2D schematic of a micromechanical model describing the velocity dependence of AE size and *b*‐value in granular fault zones. This simplistic view suggests that subparallel structures (shear bands/force chains) support the bulk of the stress and strain throughout the interseismic period. Highly stressed regions (depicted by darker particles) are separated by spectator regions (light shaded particles) that accommodate very little strain throughout the seismic cycle. Upon step increase in loading velocity, the fault zone width increases by ∆*H*. The increase in fault zone width increases fault zone porosity (decreases density) and permits the nucleation of large AEs and lower *b*‐values.

It is well established that shear within granular fault zones localizes along shear bands and that changes in fault zone dilation can be used to approximate (qualitatively) the width of the shear bands (Mair & Marone, 1999; Marone & Kilgore, 1993; Marone et al., 1990). Previous works show that shear band width decreases progressively with shear strain and that such shear localization tends to drive fault zones toward velocity weakening behavior (Marone, 1998). Our experiments are consistent with this view. Upon a step increase in slip velocity, the net fault zone thickness increases and undergoes a semipermanent net dilation, consistent with previous work (Figure 9; Mair & Marone, 1999; Marone et al., 1990; Samuelson et al., 2009; Segall & Rice, 1995). Following Marone and Kilgore (1993), we assume that fault zone dilation is a proxy for the volume of gouge material participating in shear. In other words, the volume of material participating in shear scales systematically with fault zone dilation. The increase in bulk fault zone thickness (Δ*H*_SS_) due to a step increase in slip velocity increases the porosity of the fault zone (decrease granular density) and allows for greater particle motion and the possibility for larger regions to slip in a given AE event due to the open packing between the particles (Figure 10; Samuelson et al., 2009).

We propose a micromechanical model that connects the shear velocity dependence of AE size to the bulk fault zone density/porosity (Figure 10; Samuelson et al., 2009). In particular, at low shear velocities, the fault zone width is narrow and porosity is low. This inhibits grain mobilization and promotes frictional healing between grain contact junctions. As a result, there are smaller foreshocks and higher *b*‐values prior to coseismic failure. However, the coseismic stress drop is larger because the fault zone is able to store more elastic‐strain energy via frictional healing processes (Scuderi et al., 2014, 2015). In contrast, at high shear velocities, the fault zone width is wide and fault zone porosity is high (Figure 10). This promotes grain mobilization and allows for less frictional healing to take place interseismically, which results in larger foreshocks, smaller *b*‐values, and smaller coseismic stress drops. This view is consistent with previous studies on bare‐rock surfaces, which have suggested that fault zone morphology (shear localization) regulates AE size and *b*‐values (Dresen et al., 2020; T. H. Goebel et al., 2017).

**Figure 11:**
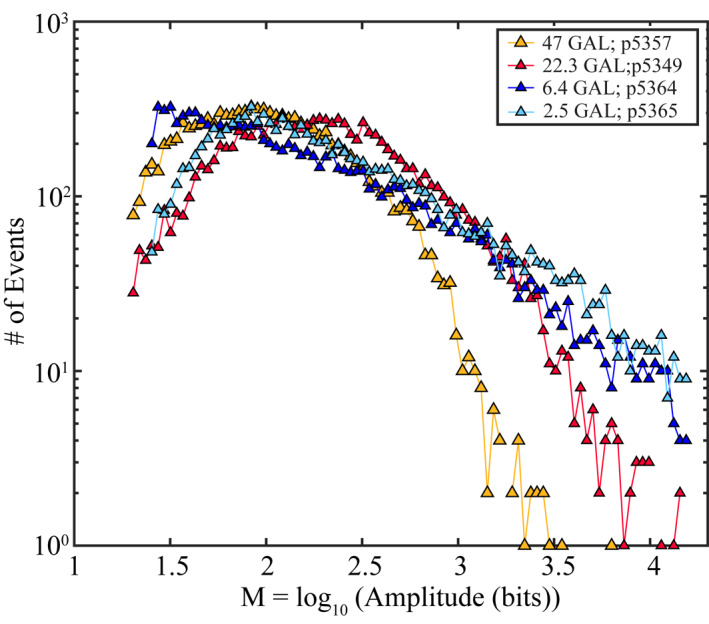
Histogram of AE amplitudes located at 85% of the peak stress for 10,000 AEs from 20 seismic cycles. The average number grains across each gouge layer (GAL) was systematically modified by varying the fault zone thickness and/or particle size (see Table 1). *F*/*M* curves show an inverse relationship between AE size and the number of grains across each gouge layer.

The proposed model above suggests that there is a connection between fault zone porosity and AE size. To test this hypothesis more directly, we conducted experiments with different particle sizes and fault zone thicknesses (Table 1). We varied particle size and fault thickness so as to vary the average number of grains across the layer (GAL) or potential force chain length (Figure 11). Granular density increases (fault zone porosity decreases) with force chain length because longer chains involve more particles with greater potential for smaller particles to occupy a void. Our data show that the tails of the noncumulative AE distributions (i.e., histograms) are systematically higher for ∼*M* ≥ 3.0 for thinner/more porous fault zones (Figure 11). Hence, foreshock magnitude scales systematically with fault zone porosity. The data of Figure 11 confirm that larger AE events are expected for high porosity fault zones, such as occur in our experiments at higher shearing velocity.

Alternatively, the data in Figure 11 and the velocity dependence of AE size could be connected to the granular inertial number, which quantifies the grain size and strain rate effects of granular materials (Taylor & Brodsky, 2017). The inertial number scales systematically with grain size and strain rate. Laboratory studies have demonstrated that granular materials with higher inertial numbers radiate more acoustic energy due to enhanced particle interactions (Taylor & Brodsky, 2017). Therefore, it is plausible that the AE characteristics of our experiments are simply modulated by granular interactions and are quantifiable by a granular inertial number.

We suggest that the velocity dependence of Δ*H*_SS_ (fault zone porosity) can explain the inverse relationship between shear velocity and *b*‐value. If the fault zone porosity is low (e.g., low shear velocities), grain motion is restricted and frictional healing processes dominate. These conditions nucleate small AEs and result in high *b*‐values. In contrast, if the fault zone porosity is high (e.g., high shear velocities), grain motion is enhanced and the destruction of contact junctions dominate, producing bigger AEs and lower *b*‐values.

#### 4.3.2 The Reduction in *b*‐value Prior to Coseismic Failure for Granular Fault Zones

The hypotheses proposed above is a simplistic view that connects fault zone porosity and grain mobilization to AE size. Higher shearing rates enhance grain mobilization, which in turn, would allow more particles (i.e., fault patches) to slip past one another. This mechanism could also explain the reduction in *b*‐value prior to failure in granular fault zones. That is, AEs that nucleate prior to coseismic failure in granular fault zones are a manifestation of the failure of multiple particles/fault patches. Bigger areas are likely to rupture closer to failure because the fault slip rate and fault zone dilation act in parallel to increase the fault zone porosity as failure approaches, which enhances grain mobilization and promotes the destruction of grain contact junctions. However, this hypothesis should be tested more thoroughly in future laboratory studies by quantifying AE source properties (e.g., source dimensions) throughout the seismic cycle.

#### 4.3.3 Scaling Up Laboratory AEs to Seismogenic Fault Zones

Previous works show that laboratory experiments coupled with AE monitoring can improve our understanding of foreshock sequences, nucleation processes, high‐frequency radiation, and source properties of tectonic earthquakes (Blanke et al., 2021; T. H. W. Goebel et al., 2013; McLaskey & Glaser, 2011; Trugman et al., 2020). For example, recent works suggest that high‐frequency seismic radiation could be connected to elastic collisions of fault zone material (Tsai & Hirth, 2020; Tsai et al., 2021). This model is consistent with laboratory data and the idea that high‐frequency AEs in laboratory experiments originate from granular processes (Bolton et al., 2020).

Laboratory foreshocks are the result of micromechanical processes acting along grain contacts with length scales on the order of microns to millimeters. In contrast, foreshocks in nature represent the rupture of much larger fault patches with length scales on the order of meters to kilometers and likely involve grain crushing and comminution, which is absent in our experiments. Furthermore, laboratory experiments involve high‐resolution measurements of fault zone and acoustic properties throughout multiple seismic cycles; such high‐resolution measurements of seismic and mechanical attributes are often unavailable at the field scale. Therefore, it is not immediately clear if and how characteristics of laboratory seismicity scale up to tectonic fault zones. At this stage, we can simply state that our laboratory experiments indicate that shearing velocity, fault slip rate, and fault zone porosity play key roles in regulating *F*/*M* statistics of lab foreshocks. Interpreting our results in light of what has been observed from previous field studies suggests that a lack of foreshock activity preceding some earthquakes could simply indicate that the fault stays locked and the fault slip rate (and preslip) is low (or nonexistent). Furthermore, it is also possible that thermal and/or chemical processes act to lithify the fault zone, thereby reducing its porosity and inhibiting any type of movement that would otherwise radiate seismic energy. This is a simplistic view that does not account for the role of secondary faults or damage zones and neglects the role of pore‐fluids (e.g., Chiarabba et al., 2020; Snell et al., 2020). Future work should focus on these aspects of foreshock dynamics. Regardless, our work highlights the importance of fault slip rate and fault zone porosity in regulating the size of foreshocks in laboratory experiments and should be carefully considered when analyzing foreshock sequences in the field.

## 5 Conclusion

We conducted shear experiments on granular fault gouge and found systematic variations in AEs as a function of time within the lab seismic cycle. We focused on AEs prior to lab earthquakes and thus these events represent foreshocks to the main stick‐slip events (lab mainshocks). We analyzed *F*/*M* statistics of lab foreshocks using a standard cataloging approach and supplemented these observations with an analysis of raw acoustic data. Statistics from the continuous acoustic records are consistent with those produced by cataloging. Our data are consistent with previous works and demonstrate that *b*‐value decreases as coseismic failure approaches. In addition to the importance of shear stress, we demonstrate that *b*‐value scales inversely with the shearing velocity and fault slip rate. We propose that the velocity dependence of AE size and *b*‐value arises from variations in fault zone porosity and grain mobilization processes. Higher shearing rates increase fault zone porosity and grain mobilization, producing bigger AEs. Our data highlights the importance of fault slip rate and fault zone porosity in unraveling the dynamics of foreshock sequences.

## Supporting information

Supporting Information S1

## Data Availability

Data in this study are publicly available at https://doi.org/10.26207/rcgg-x946.
