# Supplementary material for: Frequency‐Magnitude Statistics of Laboratory Foreshocks Vary With Shear Velocity, Fault Slip Rate, and Shear Stress
Source: J Geophys Res Solid Earth. 2021 Nov 12;126(11):e2021JB022175. doi: 10.1029/2021JB022175 (PMC9286047; doi:10.1029/2021JB022175)
Supplement: Supplementary file 1 — Supporting Information S1 [file JGRB-126-0-s001.docx]

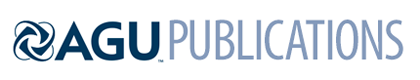


**Journal of Geophysical Research: Solid Earth**

**Supporting Information for:**

**Frequency-magnitude statistics of laboratory foreshocks vary with shear velocity, fault slip rate, and shear stress**

David C. Bolton^1*^, Srisharan Shreedharan^1^, Jacques Riviere^2^, and Chris Marone^3^

^1^University of Texas Institute for Geophysics, Austin, Texas

^2^Department of Engineering Science and Mechanics, Pennsylvania State University, University Park, Pennsylvania

^3^Dipartimento di Scienze della Terra, La Sapienza Universita di Roma, Italy

*Corresponding author: David C. Bolton ([chasbolton19@gmail.com)](mailto:chasbolton19@gmail.com))

**Contents of this File:**

Figures S1-S9

**Introduction:**

This supporting information contains figures that describe our cataloging procedure and results from our event detection and b-value sensitivity analysis. We also show plots of the cumulative number of AEs across multiple slip cycles and demonstrate how this scales with recurrence interval and inversely with shear velocity. We show how AE event rates are approximately independent of shear velocity when disregarding events with M <= M_c_. Finally, we plot F/M results from our shear stress oscillations experiments and demonstrate that F/M statistics are independent of stress state for stresses <= 50% of the peak stress.


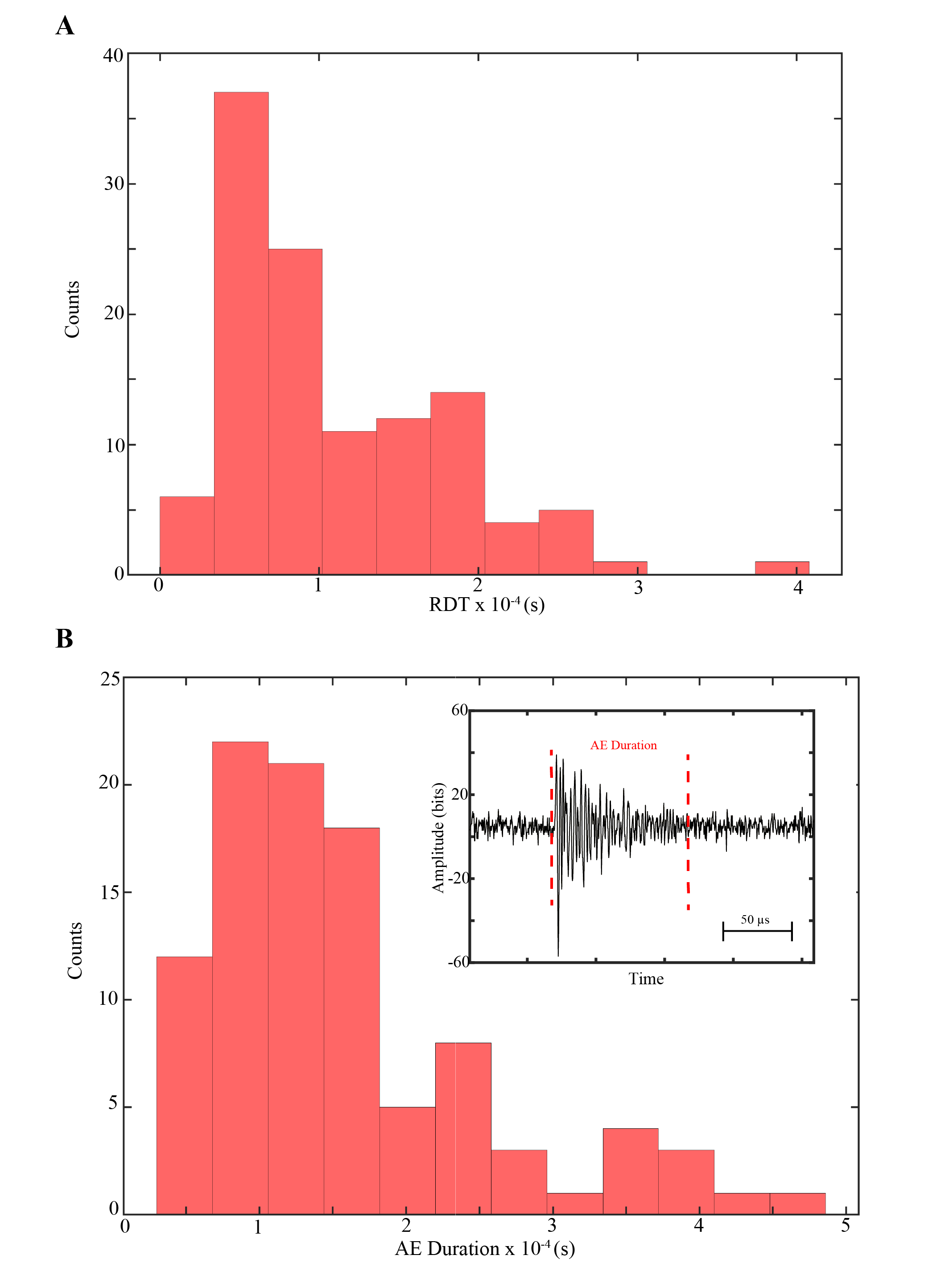


**Figure S1.** **A-B** Histogram of RDT and AE duration for several hundred AEs. Inset in B shows AE duration for a representative event. Note, the duration is derived from the raw/continuous data, and thus, is a composite of the source, sensor, and propagation effects.

**
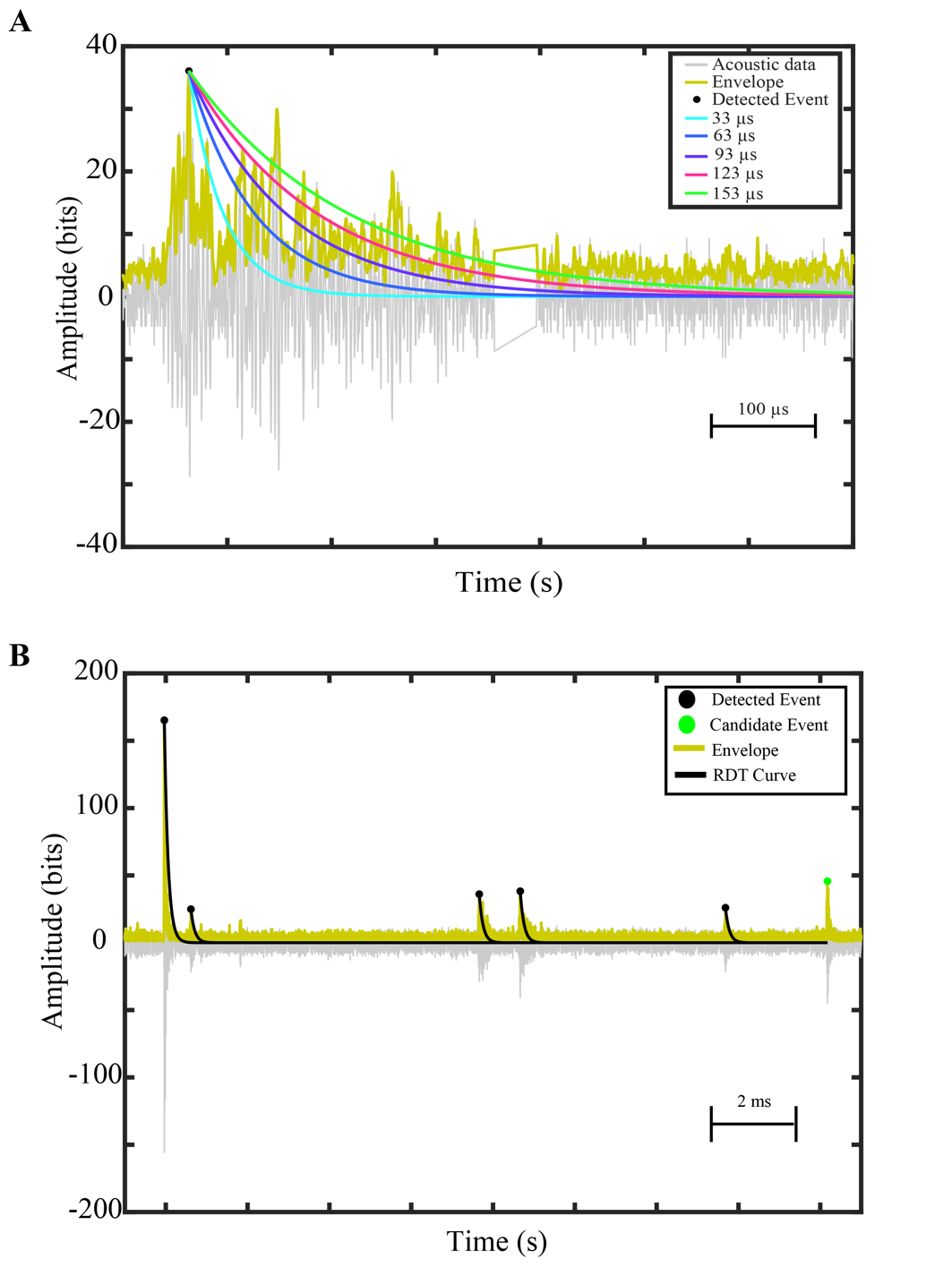
**

**Figure S2:**  **A.** Example of one AE at 3 μm/s. Superimposed on the acoustic time series data are 5 RDT curves. For this study, we use a 93 μs R.D.T to model all the AEs. **B.** Example of how the RDT parameter is implemented with a set of detected events (black symbols). Note, the event in question (candidate event) must have an amplitude that is larger than the RDT curves of the previous 5 events. In this case, the candidate event would be cataloged.

**Figure S3.** Example of how AEs are detected and cataloged using our empirical thresholding procedure (see main text for details). **A.** Raw continuous acoustic signal with 6 AEs (large spikes). **B.** Seismic signal and smoothed envelope (yellow). **C.** Seismic signal, smoothed envelope and detected AEs (black symbols). Note, the candidate events are detected after imposing a minimum amplitude (A_min_) and time threshold (T_min_) (see main text for details). **D.** Same data as panel C. Events shown in green meet the RDT threshold (Figure S1) and are cataloged. The remaining events (black symbols) are discarded from the analysis.

**
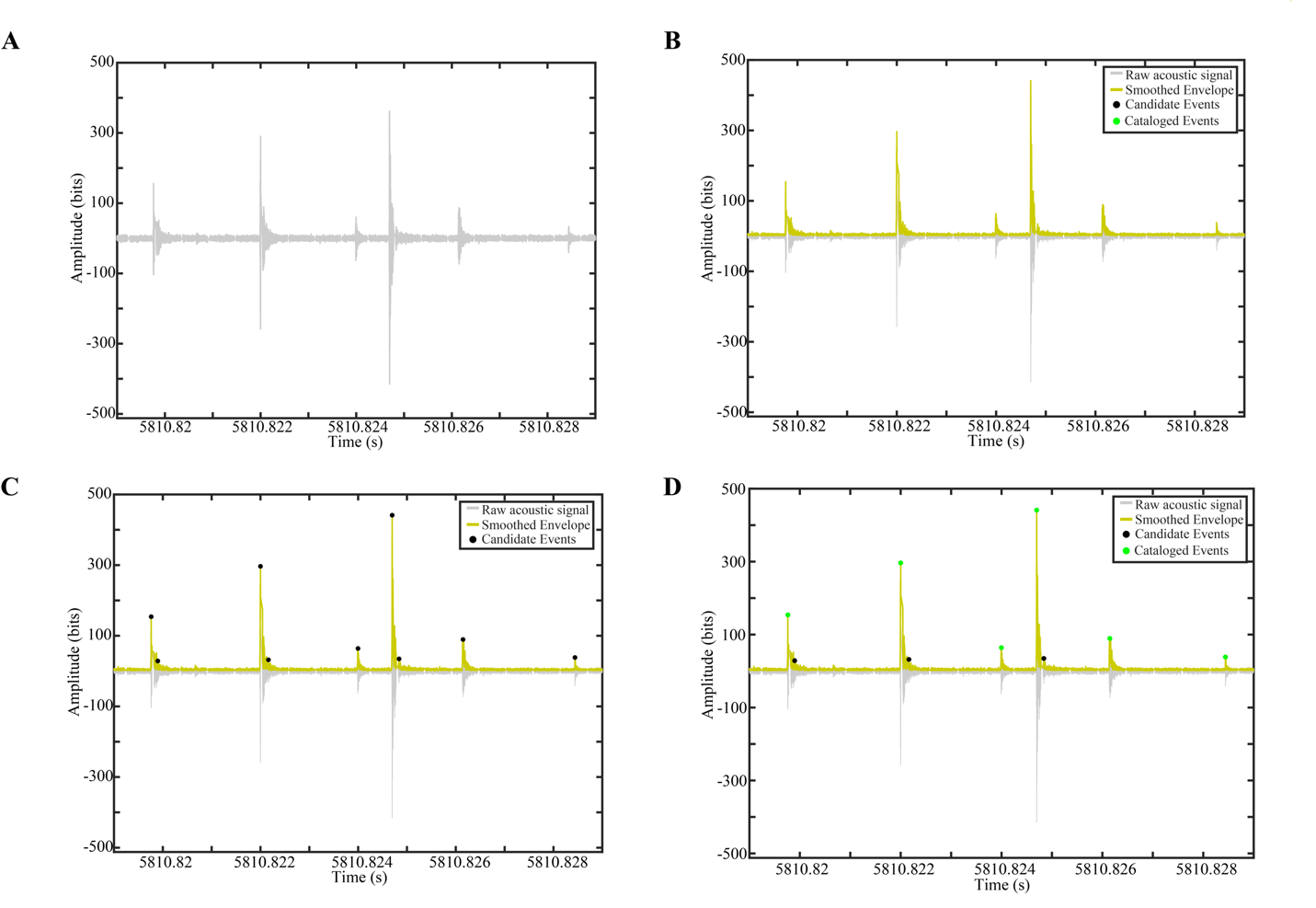
**

**
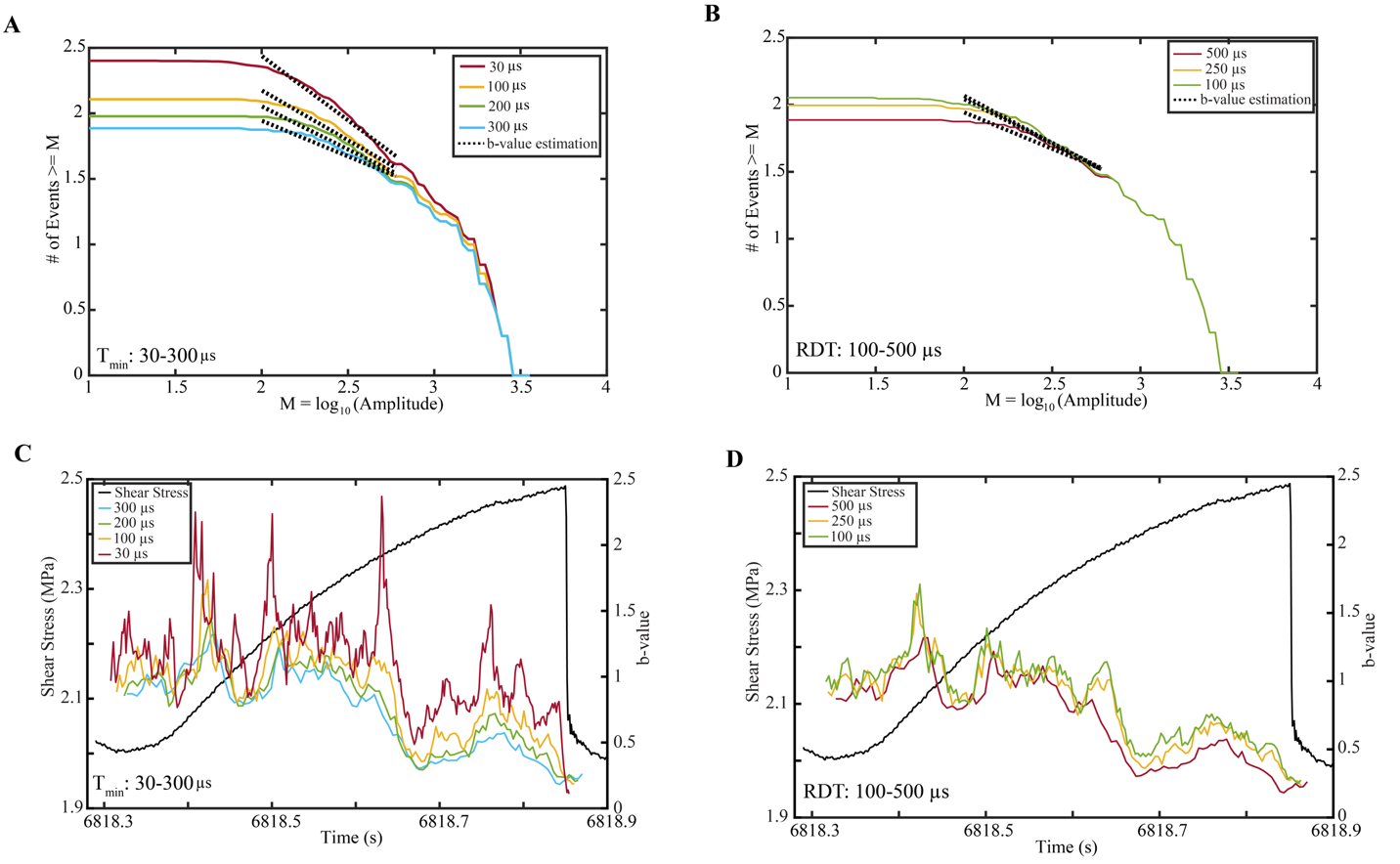
**

**Figure S4: A-B**. Frequency-magnitude curves for a range of T_min_ (A) and RDT (B) thresholds, respectively (see main text for details). The number of smaller events detected increases as T_min_ and RDT become smaller. **C-D.** Temporal evolution of b-value across one entire seismic cycle. Relative changes in b-value remain approximately the same for a wide range of T_min_ and RDT values.

**
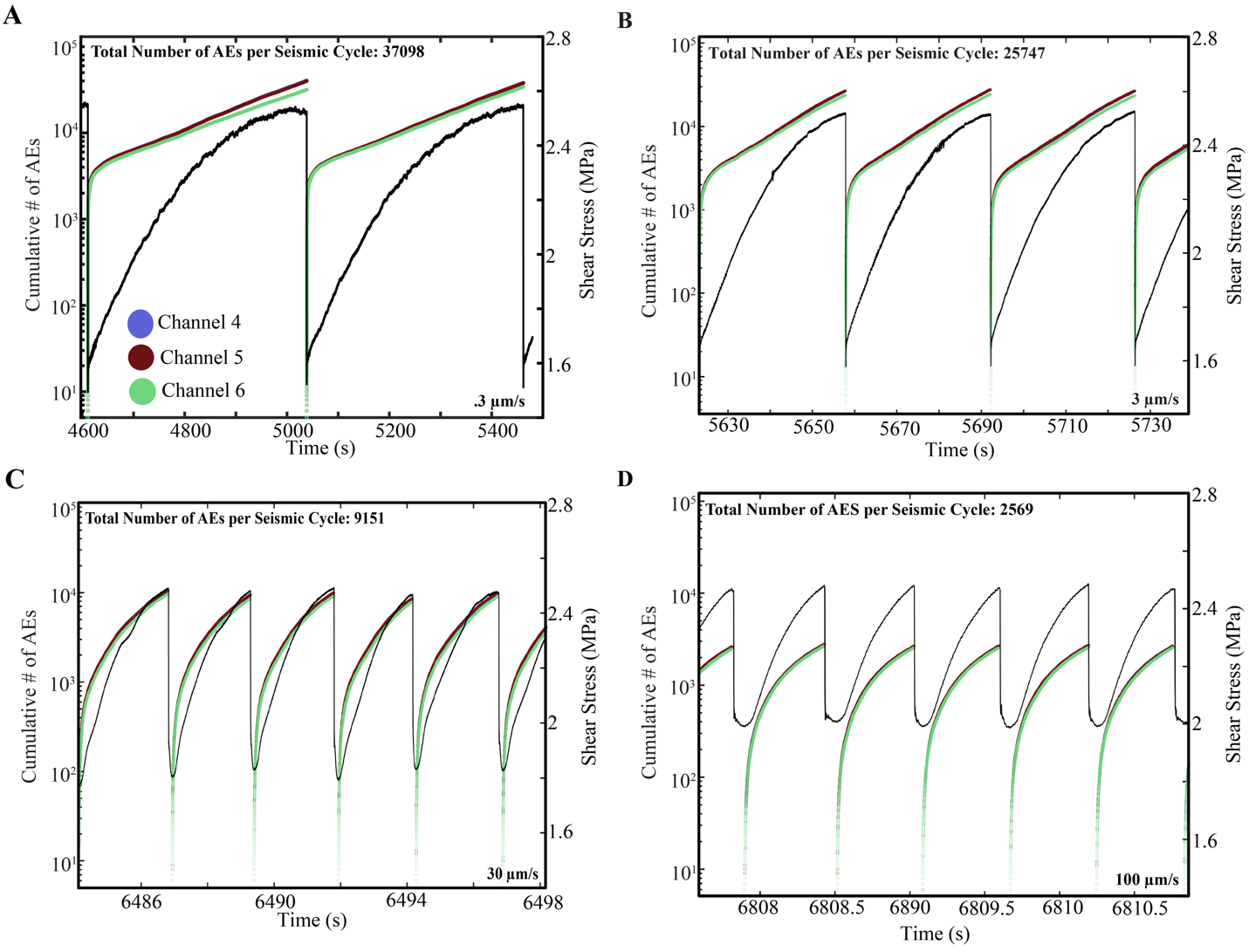
**

**Figure S5 A-D.** Cumulative number of AEs and shear stress plotted as a function of time for each shear velocity. The total number of AEs per seismic cycle scales with the recurrence interval and inversely with shear velocity. The total number of AEs used to compute b-value (see main text) corresponds to 10% of the cumulative number of AEs at a given shear velocity.

**
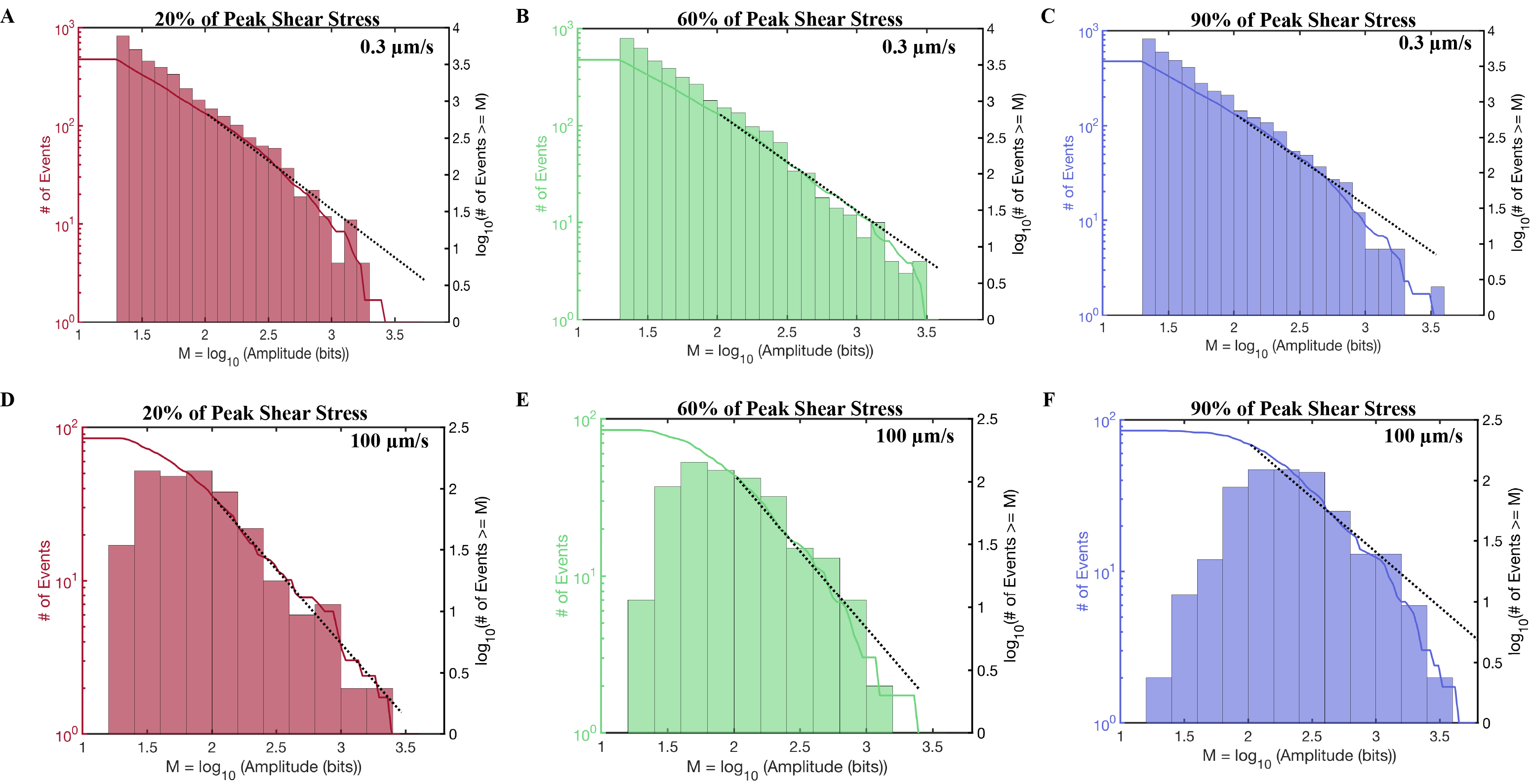
**

**Figure S6. A-C.** Cumulative (solid line) and non-cumulative (histogram) frequency-magnitude plots at different locations within the seismic cycle. Note, the F/M curves correspond to the same data shown in Figure 3 of the main text. The peak of the non-cumulative distribution corresponds to the magnitude of completeness (M_c_). M_c_ remains constant as a function of position within the seismic cycle for data at 0.3 μm/s. **D-F.** Cumulative and non-cumulative frequency-magnitude plots at different locations for the seismic cycle shown in Figure 3D. In contrast to the data at 0.3 μm/s, M_c_ shifts to higher values as failure approaches and the non-cumulative plots become more Gaussian-like and indicates that the catalog is deficient in lower magnitude events.

**Figure S7:** AE rate as a function of normalized time for data shown in Figure 2 (see main text). Note, the x-axis is scaled from the minimum shear stress to the peak shear stress for the slip cycles shown in Figure 2. AE rate is computed using the same windowing technique described in the main text, however here we only count events with the M >= 2.0. In general, the absolute value of event rates per unit shear displacement seems to be roughly independent of shear velocity for data <= 30 µm/s. Thus, the inverses relationship between event rate and shearing rate (Figure 2) could simply be due to a lack of smaller events at higher shearing velocities.

**Figure S8.**  B-value as a function of normalized slip velocity. The data plotted here correspond to the same data plotted in Figure 5. B-value scales inversely with both slip rate (low b-value at large slip velocities) and the far-field shearing rate (low b-value at large shearing rate).

**Figure S9: A.** Shear stress, AE amplitude and fault displacement plotted versus time for Experiment p5388. Initially, the fault was sheared under a constant loading rate boundary condition for ~ 10 mm. After shearing 10 mm at 21 µm/s, we reduced the shear stress on the fault to ~ 50% of the peak stress reached during the stick-slip cycles and placed a soft acrylic spring between the vertical ram and center block of the DDS to mitigate fault creep. Four series of shear stress oscillations (S1-S4) were performed at different amplitudes and frequencies that are representative of the stick-slip cycles in Experiment p5363. Amplitude and frequency of the oscillations are depicted in the left corner. The number and magnitude of AEs decreases from sequences S1 to S4. **B.** Non-cumulative frequency-magnitude data from S1 at different locations within the increasing shear stress limb. Symbols are averages across all channels and cycles in S1 at a specific location within the increasing shear stress limb and error bars represent one standard deviation. The magnitude of the AEs is approximately independent of location within the shear stress oscillation.
